# Peptide receptor radionuclide therapy in patients with medullary thyroid carcinoma: predictors and pitfalls

**DOI:** 10.1186/s12885-019-5540-5

**Published:** 2019-04-05

**Authors:** Carolien M. Beukhof, Tessa Brabander, Francien H. van Nederveen, Marie-Louise F. van Velthuysen, Yolanda B. de Rijke, Leo J. Hofland, Gaston J. H. Franssen, Lideke A. C. Fröberg, Boen L. R. Kam, W. Edward Visser, Wouter W. de Herder, Robin P. Peeters

**Affiliations:** 1 Erasmus MC, Department of Internal Medicine, Academic Center for Thyroid Diseases, European Neuroendocrine Tumor Society center of excellence, P.O Box 2040, 3000 CA Rotterdam, the Netherlands; 2 Erasmus MC, Department of Radiology & Nuclear Medicine, Erasmus University Medical Center, P.O Box 2040, 3000 CA Rotterdam, the Netherlands; 3 Erasmus MC, Department of Pathology, Erasmus University Medical Center, P.O Box 2040, 3000 CA Rotterdam, the Netherlands

**Keywords:** Thyroid cancer, medullary, Peptide receptor radionuclide therapy, Lutetium, Receptors, somatostatin

## Abstract

**Background:**

For progressive metastatic medullary thyroid carcinoma (MTC), the available treatment options with tyrosine kinase inhibitors result in grade 3–4 adverse events in a large number of patients. Peptide Receptor Radionuclide Therapy (PRRT), which has also been suggested to be a useful treatment for MTC, is usually well tolerated, but evidence on its effectivity is very limited.

**Methods:**

Retrospective evaluation of treatment effects of PRRT in a highly selected group of MTC patients, with progressive disease or refractory symptoms. In addition, a retrospective evaluation of uptake on historical ^111^In-DTPA-octreotide scans was performed in patients with detectable tumor size > 1 cm.

**Results:**

Over the last 17 years, 10 MTC patients were treated with PRRT. Four out of 10 patients showed stable disease at first follow-up (8 months after start of therapy) whereas the other 6 were progressive. Patients with stable disease were characterized by a combination of both a high uptake on ^111^In-DTPA-octreotide scan (uptake grade ≥ 3) and a positive somatostatin receptor type 2a (SSTR2a) expression of the tumor by immunohistochemistry. Retrospective evaluation of historical ^111^In-DTPA-octreotide scans of 35 non-treated MTC patients revealed low uptake (uptake grade 1) in the vast majority of patients 31/35 (89%) with intermediate uptake (uptake grade 2) in the remaining 4/35 (11%).

**Conclusions:**

PRRT using ^177^Lu-octreotate could be considered as a treatment in those patients with high uptake on ^111^In-DTPA-octreotide scan (uptake grade 3) and positive SSTR2a expression in tumor histology. Since this high uptake was present in a very limited number of patients, this treatment is only suitable in a selected group of MTC patients.

## Background

### Patients and treatment

Medullary thyroid carcinoma (MTC), originating from calcitonin (CT)-producing parafollicular C cells, is a rare form of thyroid cancer that accounts for less than 5% of thyroid carcinomas [1]. In 25% of the cases, MTC is part of inherited disorders, such as multiple endocrine neoplasia 2a, 2b or familial MTC involving RET germline mutations. Locally unresectable tumor or distant metastases have limited systemic treatment options [2, 3]. Although the tyrosine kinase inhibitors (TKI) vandetanib and cabozantinib have been shown to improve progression-free survival (PFS) [hazard ratio (HR), 0.46 and HR 0.28 respectively], grade 3 or 4 adverse events occur in a large number of patients (44% in vandetanib, 69% in cabozantinib) [3, 4]. Therefore, alternative systemic treatment options with less side effects are needed.

Somatostatin receptor (SSTR) expression has been reported in up to 85% of MTCs, particularly SSTR subtypes 2, 3 and 5 [5–8], with 49% of MTCs showing expression of the SSTR2a subtype [5]. Somatostatin receptor scintigraphy with ^111^In-DTPA-octreotide (Octreoscan®), which has high affinity for SSTR2a, has been reported to show lesional uptake in 57–65% of MTC patients [9–11]. Therefore, targeting the tumor with a radionuclide using somatostatin analogs as a ligand seems to be an attractive option.

In midgut neuroendocrine tumors, peptide receptor radionuclide therapy (PRRT) resulted in a PFS rate at 20 months of 65% vs. 11% in the control group [12].

In MTC there is limited experience with PRRT treatment. A phase II trial in 31 patients with ^90^Y-DOTATOC, which also targets SSTR2a, reported a partial response (PR) in 29% of the patients [13]. In a second trial treating 7 MTC patients with ^177^Lu-octreotate, 3 patients had PR, 3 patients had stable disease (SD) and 1 patient progressive disease (PD) [14]. These results suggest that PRRT might be a useful treatment in patients with MTC, although the total number of treated patients is very limited so far. For that reason, we performed a retrospective evaluation of treatment with ^177^Lu-octreotate in our center, where it was used in a highly selected group of 10 MTC patients with progressive disease or high risk tumor localization. In addition, we evaluated possible predictors and pitfalls of ^177^Lu-octreotate treatment in MTC.

## Methods

We retrospectively studied 10 consecutive patients with histologically proven MTC. Patients treated with ^177^Lu-octreotate between 2000 and 2017 had progressive metastatic MTC according to Response Evaluation Criteria In Solid Tumors 1.1 [15] (RECIST) or had high risk tumor localization (intracardial and compressive cervical tumor). The study was approved by the Institutional Review Board of the Erasmus Medical Center (127.545/1993/84) and written informed consent was obtained from participants.

We used the methods for ^177^Lu-octreotate therapy as described in detail previously [16]. Patients received an average of 4 cycles of ^177^Lu-octreotate, up to a cumulative dose of 27,8 to 29,6 GBq, with an interval of 6 to 10 weeks [17, 18]. Response to treatment was evaluated at a median of 8 months (3 months after the last cycle with ^177^Lu-octreotate) and subsequently at 3 monthly follow-up visits, assessing clinical, biochemical and imaging parameters. The world health organization (WHO) performance status was scored at baseline and during follow-up by the treating physician.

### End points

PFS was computed as the time from treatment initiation to progression, assessed by objective tumor response RECIST 1.1 criteria, clinical disease progression according to the treating physician, death or last documented patient visit [15]. Overall survival (OS) was computed as the time from treatment initiation to death, or until the last documented patient visit. Adverse events were scored according to Common Terminology Criteria for Adverse Events [19].

### ^111^In-DTPA-octreotide scans

**Fig. 1:**
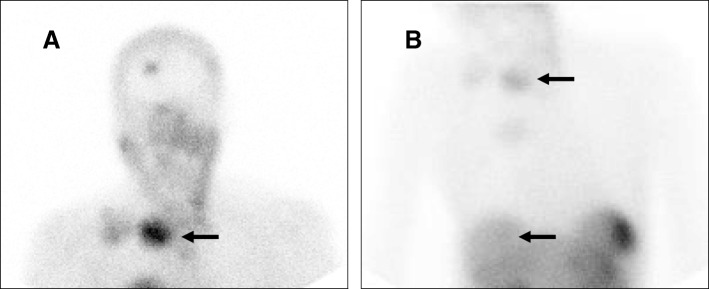
The principle of Krenning uptake on ^111^In-DTPA-octreotide scans. **a**) MTC in neck region seems to have significant uptake. **b**) However when compared to normal liver uptake it is scored as Grade 2 uptake

We retrospectively reviewed ^111^In-DTPA-octreotide scans between 1999 and 2011 of non-treated MTC patients that were performed in metastatic MTC. Although not part of the regular follow-up of MTC [2], ^111^In-DTPA-octreotide scans were performed for tumor staging in most of these patients. Uptake was scored according to the Krenning score [18, 20]: uptake grade 1 = uptake < normal liver uptake; uptake grade 2 = uptake equal to normal liver uptake; uptake grade 3 = uptake > normal liver uptake; uptake grade 4 = uptake > normal spleen or kidneys uptake (Fig. 1).

### Immunohistochemistry SSTR2a

SSTR2a immunohistochemistry was retrospectively performed in the patients treated with ^177^Lu-octreotate. Formalin fixed paraffin embedded (FFPE) tissue slices of one or two tissue biopsies per patient were immunostained for SSTR2a using the Ventana BenchMark ULTRA stainer (Ventana, Tucson, Arizona, USA), according to the protocol provided by the manufacturer. The rabbit monoclonal anti-sst2 antibody (BioTrend, Köln, Germany) was used at a dilution of 1:25. Normal pancreatic tissue served as a positive control. SSTR2a expression was scored according to the percentage of cells with positive immunohistochemistry (0: absent staining; 1: weak staining < 30% of cells; 2: moderate staining 30–60% cells; 3: strong staining> 60% of cells) and localization cytoplasm or cell membrane [21]. Scoring was done by two independent pathologists (F.H. N and M.F.V), who were blinded to each other’s findings and patient data.

### Laboratory measurements

RET gene analysis to exclude multiple endocrine neoplasia 2a, was performed in DNA of 6/10 patients. RET analysis on the tumor tissue was not performed in this retrospective study. Calcitonin (CT) and carcinoembryonic antigen (CEA) are useful tumor markers of residual disease and their doubling time is an indicator for prognosis [22]. CT was measured on an Immulite 2000XPi immunoassay system (Siemens Healthcare Diagnostics, Los Angeles, USA) by chemoluminescence method. CEA was measured by an electrochemiluminescence immunoassay on a Cobas e601 immunoassay analyzer (Roche Diagnostics GmbH, Penzberg, Germany). The inter-day coefficient of variation of CT and CEA were 7.8 and 5%, respectively.

### Statistics

We refrained from statistics due to limited patient number. PFS and OS was determined with Kaplan-Meier BM SPSS Statistics for Windows v23 (IBM Corp., Armonk, NY).

## Results

### Baseline

Ten patients were treated with ^177^Lu-octreotate. They had a median age 62 [range 19–75] years and 4/10 (40%) were male (Table 1). The indication for ^177^Lu-octreotate was progressive MTC according to RECIST-criteria in 8/10 (80%) of patients. One patient was treated for intracardial metastasis with biochemical progression and one patient for large cervical inoperable tumor load. None of the tested patients showed germline mutations in the RET proto-oncogene (Table 1). Eight out of 10 (80%) patients had a baseline WHO performance status of 1, whereas the other 2 treated patients had a WHO status of 3 (Table 1). Three out of 10 (30%) patients had an endocrine paraneoplastic syndrome due to tumor-produced adrenocorticotropic hormone (ACTH), resulting in ectopic Cushing’s disease, parathyroid hormone-related protein (PTH-rp) resulting in severe hypercalcemia and dopamine without clinical sequelae.

**Table 1:** Patient characteristics

	Overall (*N* = 10)	Stable disease (*N* = 4)	Progressive disease (*N* = 6)
Age years *median [range]*	63 [19–75]	69 [19–75]	60 [42–73]
Male N *(%)*	4/10 (40%)	1/4 (25%)	3/6 (50%)
RET			
Wild-type N (%)	6/10 (60%)	3/4 (75%)	3/6 (50%)
Unknown N (%)	4/10 (40%)	1/4 (25%)	3/6 (50%)
Disease extent			
Moderate	8/10 (80%)	2/4 (50%)	6/6 (100%)
Extensive	2/10 (20%)	2/4 (50%)	0/6 (0%)
Tumormarkers			
Calcitonin DT years, [range]	2.4 [0.6–4.1]	0.8 [0.6–4.1]	2.6 [2.4–2.9]
CEA DT years, [range]	1.9 [0.6–7.4]	1.9 [1.2–2.1]	4.0 [0.6–7.4]
PRRT indication			
PD	8/10 (70%)	3/4 (75%)	5/6 (83%)
Tumor localization^❶^	2/10 (30%)	1/4 (25%)	1/6 (17%)
WHO			
1	8/10 (80%)	3/4 (75%)	5/6 (83%)
3	2/10 (20%)	1/4 (25%)	1/6 (17%)
Hormonal functioning ^❷^	3/10 (30%)	1/4 (25%)	2/6 (33%)
Endpoints			
Death from MTC	7/10	2/4 (50%)	5/6 (83%)
Death other cause^❸^	1/10	1/4 (25%)	0/4 (0%)
Alive	2/10	1/4 (25%)	1/4 (25%)

**Abbreviations:** CEA, carcinoembryonic antigen; DT, doubling time; MTC, medullary thyroid carcinoma; N, number; PRRT, peptide receptor radionuclide therapy; RET, rearranged during transfection; WHO, world health organization performance status

**Footnote:**

❶ Intracardial metastasis and biochemical progression; inoperable cervical tumour load

❷ Ectopic ACTH; PTH-rp; dopamine

❸ Fibrosarcoma

### End points

Two patients showed PD during the third treatment cycle and were withheld from further treatment. In total, median PFS was 0.70 years [range 0.3–12.0], 1 patient is still in follow-up with stable disease 1.6 years after start of treatment. In total, 6 out of 10 (60%) patients had tumor progression at first follow-up (8 months) after start of treatment. The patient with large cervical inoperable tumor showed PD of distant metastasis, however the thyroid tumor mass with focal grade 3 expression on ^111^In-DTPA-octreotide scan remained stable.

**Fig. 2:**
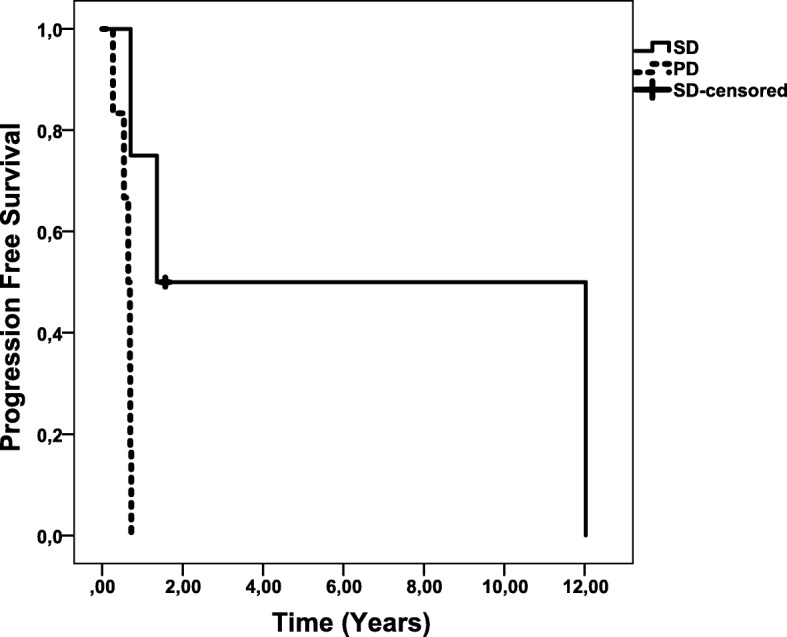
Progression free survival. Abbreviations: PD, progressive disease, SD, stable disease. Footnote: SD censored patient is still in follow-up with stable disease 1.6 years after start of treatment

Four out of 10 (40%) patients had SD at first follow-up. This includes, the patient with intracardial tumor mass with SD at start of therapy. Overall, SD was maintained for a median of 1.4 years [range 0.7–12.0] (Fig. 2). One patient with RECIST progressive disease before start of PRRT had enduring sustained SD for 12 years and died from an unrelated disease (fibrosarcoma). Overall median OS was 1.14 years [range 0.4–12.0]. Two patients are still alive at 1.4 and 1.6 years since start of PRRT. The median OS in SD patients was 1.8 years [0.8–12.0].

Baseline median CT doubling time and CEA doubling time showed a wide range making it difficult to interpret due to small patient number. Patients with SD seemed to have a trend to shorter CT and CEA doubling time than patients with PD (Table 1). None of the patients showed a decrease in CT or CEA levels of ≥50% after treatment with ^177^Lu-octreotate. Three of the four patients with SD showed a 20–40% decrease of CT and/or CEA levels with sustained response. One out of these four patients showed an initial rise of tumor markers in the first 5 months, which was followed by a sustained decrease in both tumor markers. Three out of the 6 patients with PD showed loss of correlation with CT (no increase in tumor marker, despite progressive disease) (Table 2). In one out of 6 patients there was loss of CEA tumor marker, but sustained CT response.

**Table 2:** Clinical characteristics and outcome

	Overall (N = 10)	Stable disease (N = 4)	Progressive disease (N = 6)
Uptake Grade ≥ 3	7/10 (70%)	4/4 (100%)	3/6 (50%)
Uptake Grade < 3	3/10 (30%)	0/0 (0%)	3/6 (50%)
SSTR2a tumour expression	4/10 (40%)	4/4 (100%)	0/6 (0%)
Loss of correlation CT	3/6 (50%)	0/4 (0%)	3/6 (50%)

**Abbreviations:** Calcitonin, CT; Grade 3: uptake > normal liver uptake; N, number; SSTR, somatostatin receptor

### Predictors of response

All 4 patients with SD showed high uptake on the ^111^In-DTPA-octreotide scans (uptake grade ≥ 3) and showed moderate to positive SSTR2a receptor expression on histological examination (Table 2).

**Fig. 3:**
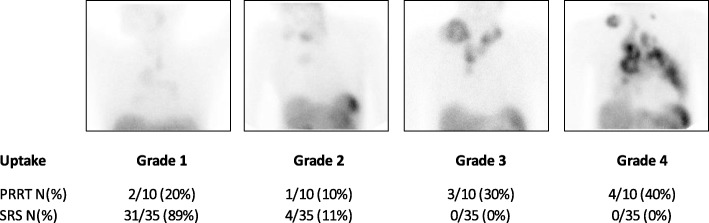
Uptake on ^111^In-DTPA-octreotide scans in patients treated with PRRT and of 35 non-treated patients with metastasized MTC. Abbreviations: N, number; PRRT, peptide receptor radionuclide therapy; SRS, somatostatin receptor scintigraphy (^111^In-DTPA-octreotide scans)

All patients with PD had negative tumor STTR2 expression in the tissue biopsy. Patients with PD had variable uptake on ^111^In-DTPA-octreotide scans, as well as on post therapy scans (Table 2). Two out of 6 patients (33%) showed uptake grade 3 on ^111^In-DTPA-octreotide scan. In one of these patients we identified a remarkable SSTR2a positive staining of tumor endothelium. In 1 out of the 6 patients (17%) non-homogenous uptake of ^111^In-DTPA-octreotide scan, with loss of uptake in some metastasis was observed. In the other patients, 1/6 (17%) showed uptake grade 2 and 2/6 (33%) showed uptake grade 1 (Fig. 3).

### ^111^In-DTPA-octreotide scans

None of the 35 retrospectively evaluated non-treated MTC patients had an ^111^In-DTPA-octreotide scan with higher uptake in the metastases than in the liver (grade ≥ 3). Only 4/35 (11%) patients showed moderate uptake (grade 2). In the majority of patients 31/35 (89%), the tumor was detectable. (Fig. 3).

### Symptoms and adverse events

There was no improvement of symptoms or paraneoplastic syndrome, neither in the stable disease patients nor in the progressive patients. Common grade 1 side effects included diarrhea, fatigue, mild anorexia and mild hair loss, occurring in the majority of patients. Grade 2 diarrhea occurred in 1 out of 10 patients. One patient developed grade 3 adverse event: hemoptysis, presumably due to progression of pulmonary metastasis.

## Discussion

This study reports the results of 10 patients with metastasized MTC that were treated with PRRT using ^177^Lu-octreotate. Only 4 patients showed SD at first follow-up at 8 months after start of therapy. All of these 4 patients with SD showed high uptake (Krenning uptake grade ≥ 3) on the ^111^In-DTPA-octreotide scan as well as SSTR2a receptor expression in the tumor upon immunohistochemistry staining, whereas none of the remaining 6 patients that had PD at first follow-up had such a combination of high uptake plus SSTR2a receptor expression in the tumor. ^177^Lu-octreotate was well tolerated, in accordance to previous publications [12].

Our results are similar to the results reported with ^90^Yttrium-labelled octreotide treatment [13], where partial response (PR) was defined as any decrease in tumor marker. In the present study this was identified in 3 out of 10 (30%) patients. Moreover, median OS was in a similar range, i.e. 1.3 years in patients treated with ^90^Yttrium-labelled octreotide vs. 1.14 years in our study population. However, ^90Yttrium^-labelled octreotide is known to cause more severe hematological and renal side effects [13].

Although patient numbers are small, the study by Vaisman [14], suggests a better treatment response compared to the present study, where 6/10 (60%) patients had PD. PFS was 6.38 years in the study by Vasiman et al. versus 0.70 years in our patient population. This suggests that our study population included patients with more aggressive MTC. Several other study characteristics support this hypothesis. Firstly, the present study included 3/10 (30%) patients with paraneoplastic endocrine syndrome, which is a well-known poor prognostic factor [23]. Secondly 4/10 (40%) patients showed loss of tumor marker expression, which is also associated with worse prognosis [24]. And finally, our patients were much older (median age of 62 years [range 19–75] in the current study versus 35 years [range 20–78] in the study by Vaisman et al.), which is relevant, as age is an important determinant of prognosis in MTC [25].

The hypothesis that our study population had an aggressive form of MTC is also supported by the very low PFS in the present study, 0.7 years compared to 1.6 years in the placebo group of the phase III trial of the ZETA study (vandetanib) [4].

### Predictors of response

All 4 patients with SD were characterized by high uptake on ^111^In-DTPA-octreotide scan (grade ≥ 3) and SSTR2a tumor expression.

In the previously mentioned trial with ^90^Yttrium-labelled octreotide [14], tumor response did not correlate with ^111^In-DTPA-octreotide uptake. In that study only 2 out of 9 (22%) of the responders had a high uptake (grade > 3) vs. 6 out of 22 (27%) of the non-responders. However, response was based on tumor marker decrease and not scored according to RECIST criteria. SSTR2a staining on the tumor specimen was not performed.

Retrospective evaluation of historical ^111^In-DTPA-octreotide scans shows that only very few MTC patients have ^111^In-DTPA-octreotide scans with uptake grade ≥ 3. Other papers have reported uptake in up to 65% of MTC patients, but none of these studies reported a formal uptake grade with comparison to hepatic uptake [9, 10].

Despite uptake grade ≥ 3 on ^111^In-DTPA-octreotide scan, we identified 3/6 (50%) patients in the PD group who had negative SSTR2a tumor staining upon immunohistochemistry. This might be explained by non-homogenous tumor expression of the SSTR2a receptor. Moreover, in one of these patients with PD, we identified SSTR2a immunohistochemistry uptake in the vascular endothelium instead of in the tumor itself, which may have resulted in uptake on ^111^In-DTPA-octreotide scan. This phenomenon is described in a variety of other human tumors [26, 27].

In the present study SSTR2a expression was absent in all 6 patients with PD, which suggests that lack of SSTR2a expression in the tissue biopsy may be a bad prognostic sign in patients with MTC. This is in line with pancreatic NET (P-NETs) and GEP-NETs, where low SSTR2a tumor expression has been shown to be associated with poor outcome and more aggressive grades of tumor [28–30]. In a retrospective study in 97 patients with MTC, SSTR2a expression was significantly correlated with the presence of lymph node metastasis. However, prognosis was not investigated [8]. In MTC patients, stage IV, 10-year survival rates for SSTR2a negative patients was 43% versus 96% for SSTR2a positive patients [31].

### Limitations and future perspectives

A limitation of the present retrospective study is the small number of patients and the heterogeneity between patients. Despite this, as very limited data of PRRT in MTC patients are currently available, the present study provides valuable insights about which patients might potentially benefit from PRRT. In addition, it clearly illustrates that this therapy may only be suitable for a highly selected group of patients.

In the present study, 111In-DTPA-octreotide scans were used to evaluate SSTR2a uptake in 35 non-treated patients, demonstrating very limited uptake compared to the liver in the majority of patients. The nowadays more commonly used 68Ga-DOTATATE PET scans have better imaging properties due to pharmacological (higher affinity to SSTR2a), technical (e.g. positron imaging, attenuation correction), as well as physical (higher gamma energies) differences and are more useful for more precise staging of the patient [32–34]. However, it is not likely that routine 68Ga-DOTATATE PET scans would have resulted in a higher percentage of patients with a more favorable uptake compared to the liver. In pulmonary and gastroenteropancreatic neuroendocrine tumours only 3 out of 78 (4%) patients were misclassified by 111In-DTPA-octreotide scans [35].

The role of other pharmaceuticals in imaging and treatment of MTC, such as metaiodobenzylguanidine (MIBG), is limited due to low sensitivity (30%) [36, 37] and the small number of patients treated with I-131-MIBG [38, 39].

## Conclusion

PRRT using ^177^Lu-octreotate could be considered in MTC patients with both a high tumor uptake (≥grade 3) on ^111^In-DTPA-octreotide scan as well as tumor SSTR2a receptor expression by immunohistochemistry. Further research is needed to evaluate the effectiveness in these patients. Our retrospective data of 35 non-treated MTC patients, suggest that only minority of patients are eligible for ^177^Lu-octreotate therapy.

## Abbreviations

CEA: carcinoembryonic antigen
CI: confidence interval
CT: Calcitonin
GEP-NETs: gastroenteropancreatic neuroendocrine tumors
HR: hazard ratio
MIBG: metaiodobenzylguanidine
MTC: medullary thyroid carcinoma
OS: Overall Survival
PD: progressive disease
PFS: progression-free survival
P-NETs: pancreatic neuroendocrine tumors
PR: partial response
PRRT: peptide receptor radionuclide therapy
RECIST: Response Evaluation Criteria In Solid Tumors
RET: rearranged during transfection
SD: stable disease
SSTR: somatostatin receptor
TKI: Tyrosine kinase inhibitor
uptake grade 1: uptake < normal liver uptake
uptake grade 2: uptake equal to normal liver uptake
uptake grade 3: uptake > normal liver uptake
uptake grade 4: uptake > normal spleen or kidneys uptake
WHO: world health organization
